# Muscle Structure Influences Utrophin Expression in *mdx* Mice

**DOI:** 10.1371/journal.pgen.1004431

**Published:** 2014-06-12

**Authors:** Glen B. Banks, Ariana C. Combs, Guy L. Odom, Robert J. Bloch, Jeffrey S. Chamberlain

**Affiliations:** 1 Department of Neurology, University of Washington, Seattle, Washington, United States of America; 2 Department of Physiology, University of Maryland School of Medicine, Baltimore, Maryland, United States of America; The Jackson Laboratory, United States of America

## Abstract

Duchenne muscular dystrophy (DMD) is a severe muscle wasting disorder caused by mutations in the dystrophin gene. To examine the influence of muscle structure on the pathogenesis of DMD we generated *mdx^4cv^*:desmin double knockout (dko) mice. The dko male mice died of apparent cardiorespiratory failure at a median age of 76 days compared to 609 days for the desmin^−/−^ mice. An ∼2.5 fold increase in utrophin expression in the dko skeletal muscles prevented necrosis in ∼91% of 1a, 2a and 2d/x fiber-types. In contrast, utrophin expression was reduced in the extrasynaptic sarcolemma of the dko fast 2b fibers leading to increased membrane fragility and dystrophic pathology. Despite lacking extrasynaptic utrophin, the dko fast 2b fibers were less dystrophic than the *mdx^4cv^* fast 2b fibers suggesting utrophin-independent mechanisms were also contributing to the reduced dystrophic pathology. We found no overt change in the regenerative capacity of muscle stem cells when comparing the wild-type, desmin^−/−^, *mdx^4cv^* and dko gastrocnemius muscles injured with notexin. Utrophin could form costameric striations with α-sarcomeric actin in the dko to maintain the integrity of the membrane, but the lack of restoration of the NODS (nNOS, α-dystrobrevin 1 and 2, α1-syntrophin) complex and desmin coincided with profound changes to the sarcomere alignment in the diaphragm, deposition of collagen between the myofibers, and impaired diaphragm function. We conclude that the dko mice may provide new insights into the structural mechanisms that influence endogenous utrophin expression that are pertinent for developing a therapy for DMD.

## Author Summary

Duchenne muscular dystrophy (DMD) is a severe muscle wasting disorder caused by mutations in the dystrophin gene. Utrophin is structurally similar to dystrophin and improving its expression can prevent skeletal muscle necrosis in the *mdx* mouse model of DMD. Consequently, improving utrophin expression is a primary therapeutic target for treating DMD. While the downstream mechanisms that influence utrophin expression and stability are well described, the upstream mechanisms are less clear. Here, we found that perturbing the highly ordered structure of striated muscle by genetically deleting desmin from *mdx* mice increased utrophin expression to levels that prevented skeletal muscle necrosis. Thus, the *mdx*:desmin double knockout mice may prove valuable in determining the upstream mechanisms that influence utrophin expression to develop a therapy for DMD.

## Introduction

Duchenne muscular dystrophy (DMD) is an X-linked muscle disorder that affects approximately 1∶4000 boys [1]. DMD is caused by mutations in the large 2.2 Mb dystrophin gene [2], [3]. The dystrophin protein functions as a large molecular spring that connects the skeletal muscle cytoskeleton to the transmembrane dystrophin glycoprotein complex (DGC) [4]–[9]. The lack of dystrophin in DMD is accompanied by a significant reduction in the expression of the DGC leaving the membrane highly susceptible to contraction-induced injury and hypoxic stress [10]–[18]. DMD patients develop severe cardiorespiratory distress and generally live into their third decade with the help of palliative care.

The absence of dystrophin leads to various molecular and cellular homeostatic responses that slow the loss of skeletal muscle [19]. For instance, the dystrophin paralog, utrophin is expressed on the sarcolemma of dystrophic fibers acting to mitigate necrosis [20]–[25]. Skeletal muscle necrosis in the *mdx* mouse model of DMD is prevented by the expression of a full-length utrophin transgene when expressed at twice the levels of the endogenous utrophin [26]. Utrophin expression in DMD patients correlates with the severity of disease and time to wheelchair demonstrating the therapeutic potential of utrophin in humans [25], [27]–[31]. An utrophin therapy would benefit all DMD patients and circumvent a potential T-cell mediated immune response that could impair the long-term benefit of prospective dystrophin replacement strategies [32]–[34]. Accordingly, increasing the expression of utrophin is a primary target for therapy of DMD [33]. While promising utrophin-mediated therapies are being tested in clinical trials [33], [35], the mechanisms that influence utrophin expression are not fully understood.

Utrophin is normally expressed on the sarcolemma of developing and regenerating muscle fibers [21], [22], [36]. Utrophin is ultimately replaced by dystrophin in the sarcolemma of normal maturing fibers and remains concentrated at the neuromuscular and myotendinous junctions. However, low levels of utrophin can remain on the sarcolemma of dystrophin-deficient *mdx* mouse skeletal muscle fibers independent from muscle regeneration [37]. While various factors that influence utrophin expression and stability within the sarcolemma are well described [33], [38], [39], the upstream mechanisms are less clear. We recently discovered an increase in utrophin expression in *mdx^4cv^* mice expressing the microdystrophin^ΔR4–R23^ transgene [40]. The polyproline site within hinge 2 of microdystrophin^ΔR4–R23^ led to myotendinous strain injury and the formation of ringed fibers where the peripheral sarcomeres surround the central sarcomeres [40], [41]. Notably, we found a significant increase in utrophin expression within the limb muscles that contained ringed fibers, but not in the diaphragm muscles that did not contain ringed fibers [40]. Accordingly, we hypothesize that structural changes within skeletal muscle can influence utrophin expression, independent from muscle regeneration.

To examine the role of muscle structure on the pathogenesis of DMD we generated *mdx*:desmin double knockout (dko) mice. Desmin is an intermediate filament protein that maintains the highly ordered structure of striated muscles by connecting the sarcomeres to the sarcolemma and organelles [42]–[45]. Desmin influences the organization of dystrophin and ankyrin in a costameric lattice that connects the Z-disks of peripheral sarcomeres to the sarcolemma [46], [47]. Desmin^−/−^ mice develop a severe dilated cardiomyopathy with a mild skeletal myopathy [45], [48]. The skeletal myopathy is associated with misaligned sarcomeres and changes to the distribution and function of mitochondria [45], [48]. Here, we report that a ∼2.5-fold increase in utrophin expression in dko skeletal muscle fibers prevented necrosis in a fiber-type specific manner.

## Results

### Premature death of dko mice

**Figure 1:**
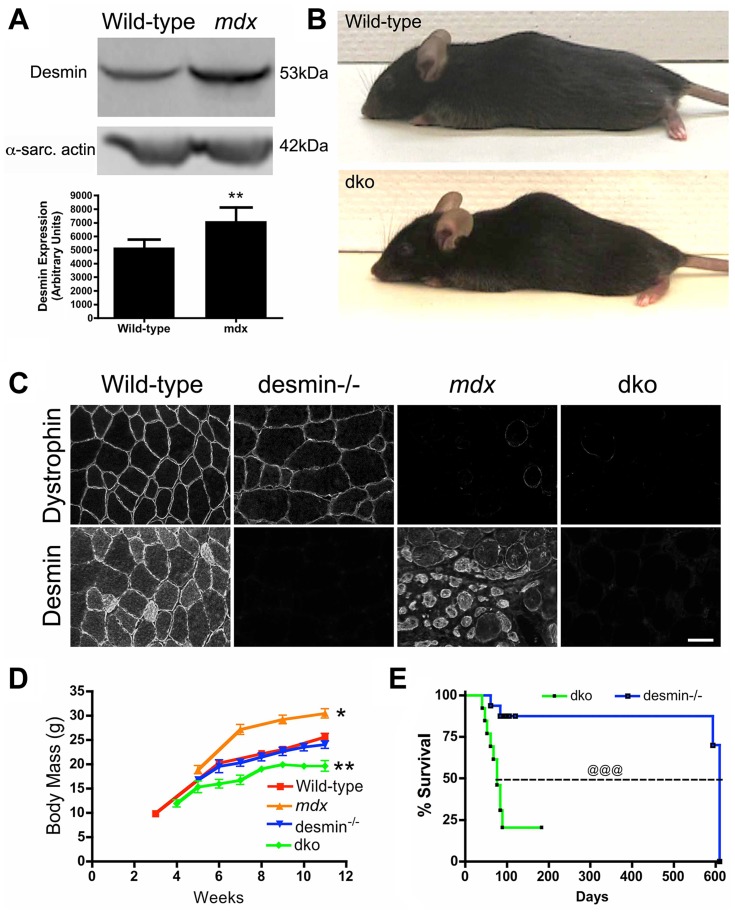
The genetic deletion of desmin from *mdx^4cv^* mice reduces body mass and survival. **A**) Desmin expression was significantly increased in the *mdx^4cv^* skeletal muscles. Bars represent the mean +/− S.D. densitometry of desmin expression from n = 7 wild-type and n = 6 *mdx^4cv^* gastrocnemius muscles. **B**) Photograph of representative wild-type and dko mice. Note that the dko mice develop a mild kyphosis at 11 weeks of age. **C**) Confirmation of genotype by immunostaining of frozen gastrocnemius muscle sections with antibodies to dystrophin and desmin. Scale bar  = 50 µm **D**) Mean +/− S.D. body mass of wild-type (n = 5), *mdx^4cv^* (n = 4), desmin^−/−^ (n = 5) and dko mice (n = 9). **E**) Kaplan-Meyer survival analyses demonstrating the significantly shortened lifespan of dko mice (n = 13) compared with desmin^−/−^ mice (n = 16). *P<0.05, **P<0.01 compared with wild-type. ^@@@^P<0.001 compared to desmin^−/−^.

We initially found that desmin expression was increased in *mdx^4cv^* mouse skeletal muscles by western analysis of whole muscle lysates (Fig. 1A), confirming previous reports in *mdx* mice [49], [50]. To examine the role of desmin in the pathogenesis of DMD we bred *mdx^4cv^*:desmin^+/−^ mice to generate the dko pups (N = 5, F>4). The dko pups were born in the expected Mendelian ratios [71 (25%) +/+; 144 (51%) +/−; 67 (24%) −/−]. We examined only the male mice for this study, as DMD patients are males. The dko mice developed a mild kyphosis (Fig. 1B). The genotype was confirmed by immunohistological analyses of dystrophin and desmin expression in skeletal muscle (Fig. 1C). The dko mice gained less body mass than the wild-type (24%), desmin^−/−^ (18%), and *mdx^4cv^* controls (36%; *P*<0.001 one way ANOVA; Fig. 1D). The desmin^−/−^ and dko mice were euthanized when they lost body mass and/or exhibited labored breathing and reduced mobility consistent with cardiorespiratory failure. Kaplan-Meyer survival analyses demonstrated a significantly reduced lifespan in the dko mice with a median survival of 76 days for males compared to a median survival of 609 days for the desmin^−/−^ males (Fig. 1E, *P*<0.001). The average lifespan for *mdx^4cv^* males is 21.5 months [51]. We chose a time point of 11 weeks for the experiments in this study, unless otherwise stated. Approximately a quarter of the dko mice (22%) developed malocclusion, which contributed to the reduced body mass and increased mortality rate particularly in mice younger than 8 weeks of age. The malocclusion was treated with trimming the teeth every week and feeding the mice crushed food pellets mixed with hydrated gel. Malocclusion consistently presented in dko mice through various backcrosses suggesting that this was likely a phenotype of the dko mice and not a separate genetic defect. Furthermore, none of the wild-type, desmin^−/−^ or *mdx^4cv^* mice developed malocclusion during the course of this study. The dko mice that developed malocclusion were included for body mass and survival analysis, but not for further analyses.

### Profound reduction in dystrophic histopathology in dko mice

**Figure 2:**
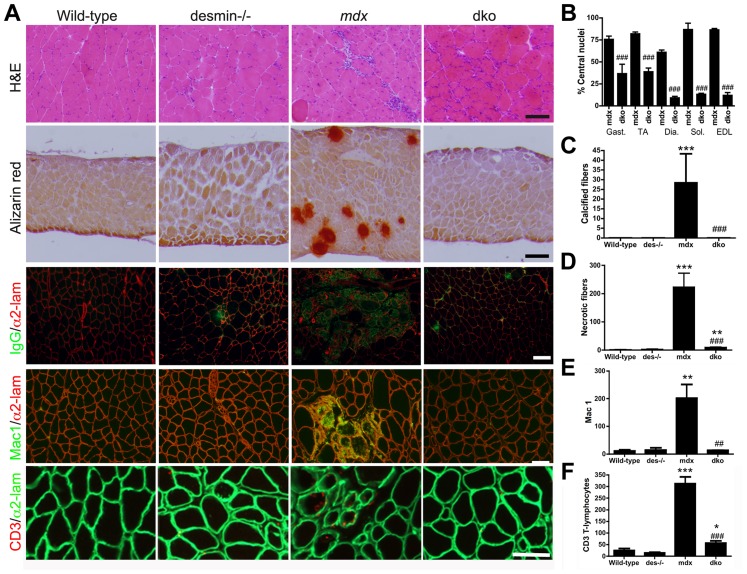
The dystrophic histopathology in *mdx^4cv^* mice was profoundly improved by the absence of desmin. **A**) Frozen sections demonstrating the histopathology of skeletal muscles. Note that the extensive central nucleation and mononuclear cell infiltrate, calcification, necrosis and inflammation in *mdx^4cv^* muscles were significantly diminished in the dko skeletal muscles. All panels are representative sections of gastrocnemius muscle except the second row, which are sections of the diaphragm. Scale bars  = 100 µm. **B**) The number of centrally nucleated fibers was significantly diminished in hind-limb and respiratory muscles in the dko mice when compared with the *mdx^4cv^* muscles **C**) Calcified fibers were found in the *mdx^4cv^* diaphragm muscle but not in the dko muscles. **D**) Quantitation of the total number of necrotic fibers in the gastrocnemius muscles. **E**) Quantitation of macrophages in the gastrocnemius muscles. **F**) Quantitation of the CD3 positive T-lymphocytes in the gastrocnemius muscles. N = 4 for all experiments. All bars in the graphs represent mean +/− S.D. *P<0.05, **P<0.01 and ***P<0.001 compared to wild-type; ^#^ *P*<0.05, ^##^ *P*<0.01 and ^###^ *P*<0.001 compared to *mdx^4cv^*.

We next examined the gross dystrophic histopathology in various limb and respiratory muscles. Wild-type mice had few central nuclei (<1%), and no detectable calcified or necrotic fibers (Fig. 2A-D). Desmin^−/−^ mice had a mild skeletal myopathy with a low level of central nuclei (∼5%) and rare necrotic fibers (Fig. 2AD), but no calcification was evident (Fig. 2A,C), as previously described [45], [48], [52]. The *mdx^4cv^* skeletal muscles were highly dystrophic with predominantly centrally nucleated fibers (Fig. 2A,B). Of the different limb and respiratory muscles we examined, only the *mdx^4cv^* diaphragms consistently contained calcified fibers (Fig. 2A,C), whereas all *mdx^4cv^* muscles contained patches of necrotic fibers (Fig. 2A,D). The proportion of dko limb and respiratory skeletal muscles with central nuclei was significantly reduced when compared to the *mdx^4cv^* muscles (Fig. 2A,B). None of the dko skeletal muscle fibers were calcified and there were 96% fewer necrotic fibers than the *mdx^4cv^* gastrocnemius muscles (*P*<0.001; Fig. 2A,C,D). Inflammation was also reduced in the dko gastrocnemius muscle with a 93% reduction in macrophages (*P*<0.01; Fig 2A,E) and an 82% reduction in CD3 positive T-lymphocytes (*P*<0.001; Fig. 2A,F) when compared to the *mdx^4cv^* controls. Thus, multiple indices of dystrophic histopathology in the *mdx^4cv^* mice were improved by the absence of desmin.

### Increased expression of utrophin prevented necrosis of dko skeletal muscle fibers

**Figure 3:**
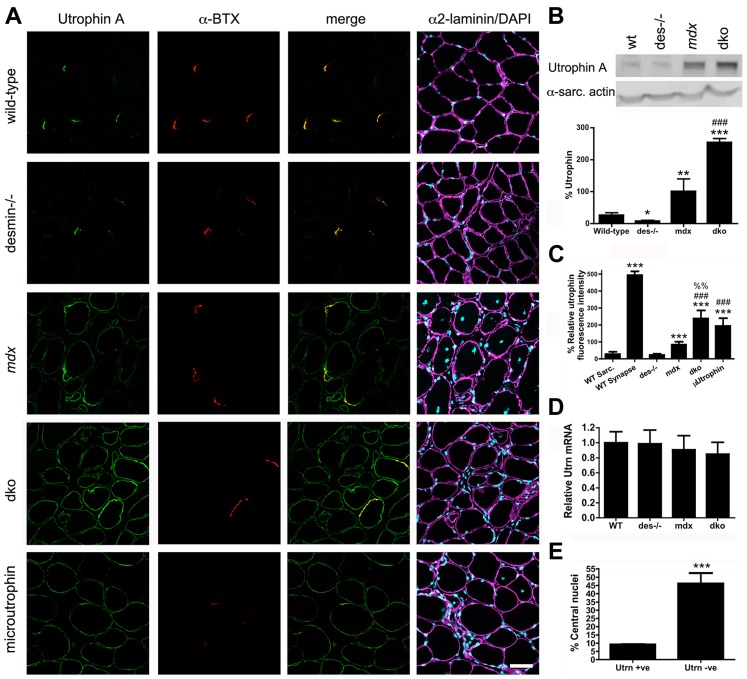
Expression and localization of utrophin in wild-type, desmin^−/−^, *mdx^4cv^* and dko muscles at 11 weeks of age. **A**) Frozen sections of the gastrocnemius muscles immunolabelled with antibodies to utrophin A and α-bungarotoxin (α-BTX). Utrophin was restricted to the neuromuscular junctions in wild-type and desmin^−/−^ muscles. Utrophin was expressed on the extrasynaptic sarcolemma in the *mdx^4cv^* and dko muscles. Note the increased utrophin expression on the sarcolemma of dko fibers compared with the *mdx^4cv^* muscles. Scale bar  = 50 µm. **B**) Western blot analyses of utrophin A expression in whole gastrocnemius muscle lysates from wild-type (n = 3), desmin^−/−^ (n = 3), *mdx^4cv^* (n = 7) and dko (n = 6) mice. Quantitation of utrophin expression in whole muscle lysates is shown below the immunoblots. **C**) Maximal utrophin fluorescence intensity was significantly increased on the sarcolemma of dko fibers compared with *mdx^4cv^* fibers. Furthermore, maximal fluorescence intensity was significantly increased in dko fibers compared to *mdx*:utrophin double knockout fibers expressing microutrophin^ΔR4–R21^. N = 4. **D**) We found no change in utrophin mRNA when comparing whole gastrocnemius muscle lysates when utrophin mRNA was normalized to the housekeeping gene Ywhaz. N = 4. **E**) Utrophin prevents muscle degeneration and regeneration in dko gastrocnemius muscles as demonstrated by the reduced proportion of fibers with central nuclei. N = 4. All bar graphs show the mean +/− S.D. *P<0.05 and ***P<0.001 compared to wild-type; ^#^ *P*<0.05 and ^###^ *P*<0.001 compared to *mdx^4cv^*.

We next examined whether the dystrophic pathology in the dko muscles was improved by an increase in utrophin expression. We examined the gastrocnemius muscle because of its distinct fiber-type distribution. Utrophin was restricted to the neuromuscular junctions in mature (11 week) wild-type and desmin^−/−^ skeletal muscle fibers (Fig. 3A). Utrophin was expressed at low levels on the extrasynaptic sarcolemma in *mdx^4cv^* muscles (Fig. 3A), as previously described in *mdx* mice [21], [22], [36]. Utrophin was highly expressed in the dko extrasynaptic sarcolemma in some, but not all of the gastrocnemius muscle fibers (Fig. 3A). We next performed a titration of dko utrophin by western analyses to generate a non-linear regression to quantitate the changes in utrophin expression (Fig. S1). The significant increase in utrophin expression in *mdx^4cv^* mice compared to wild-type mice was confirmed by western analysis of total gastrocnemius muscle lysates (Fig. 3B; *P*<0.001). Importantly, we found a 2.54-fold increase in utrophin expression in the dko when compared with the *mdx^4cv^* controls (Fig. 3B; *P*<0.001). Because not all myofibers express utrophin in the dko we next quantitated the level of utrophin fluorescence intensity on the sarcolemma. We quantitated utrophin fluorescence in the wild-type sarcolemma as the negative control and the wild-type neuromuscular synapse as the peak of detection to ensure our quantitation is not beyond the limits of detection. The fluorescence intensity of utrophin was significantly increased in *mdx^4cv^* muscles compared to wild-type muscles (*P*<0.001; Fig. 3C). The utrophin fluorescence intensity increased by 2.86-fold in the dko sarcolemma when compared to the *mdx^4cv^* (*P*<0.001). To test whether this increase in fluorescence intensity in the dko reached therapeutic levels, we compared *mdx*:utrophin double knockout muscles treated with microutrophin^ΔR4–R21^ using the same gastrocnemius muscles from our previous study [53], which demonstrated that microutrophin^ΔR4–R21^ prevented skeletal muscle necrosis. We found that the sarcolemmal fluorescence intensity of utrophin was increased by 22% in the dko muscles when compared to the *mdx*:utrophin double knockout muscles expressing microutrophin^ΔR4–R21^ (*P*<0.01). We found no change in utrophin mRNA in the gastrocnemius muscles of wild-type, desmin^−/−^, *mdx^4cv^* and dko mice, when measured by qPCR (Fig. 3D). Upregulation of utrophin was associated with a reduction in necrosis and regeneration in the dko, as only 9% of the fibers with extrasynaptic utrophin had central nuclei compared with 46% central nuclei in fibers without extrasynaptic utrophin (*P*<0.001; Fig. 3E). Thus, an increase in utrophin expression in a fraction of the dko muscle fibers prevented cycles of necrosis and regeneration.

### Utrophin expression on the sarcolemma of maturing 1a, 2a and 2d/x fiber types

**Figure 4:**
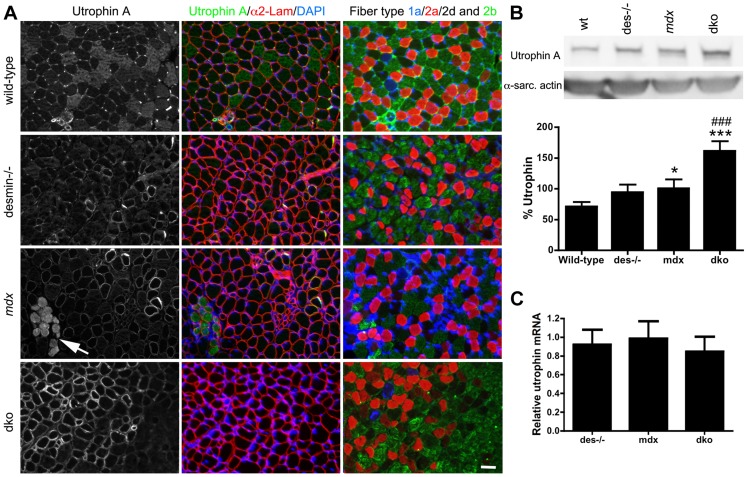
Expression and localization of utrophin in wild-type, desmin^−/−^, *mdx^4cv^* and dko muscles at 3 weeks of age. **A**) Utrophin expression in gastrocnemius muscles compared to adjacent sections labeled for the different skeletal muscle fiber-types. Note that utrophin expression is restricted to the neuromuscular junctions and non-muscle cells in the wild-type muscles. Utrophin is also restricted to the neuromuscular junctions in most fast 2b fibers in desmin^−/−^ muscles, but remains on the sarcolemma of the type 1a, 2a and 2d/x fiber types. Utrophin is found on the extrasynaptic sarcolemma of *mdx^4cv^* muscles, irrespective of fiber-type at 3 weeks of age. Regions where utrophin is lost from the extrasynaptic sarcolemma in *mdx^4cv^* muscles have necrotic fibers (arrows). Utrophin expression is lost from most of the fast 2b fibers in the dko by 3 weeks of age, but is retained on the sarcolemma of 1a, 2a and 2d/x fiber types. Scale bar  = 50 µm. **B**) Western blot analyses of utrophin A expression in whole gastrocnemius muscle lysates from (n = 4), desmin^−/−^ (n = 4), *mdx^4cv^* (n = 8) and dko (n = 8) mice. Quantitation of utrophin expression in whole muscle lysates is shown below the immunoblots. C) We found no change in utrophin mRNA when comparing whole gastrocnemius muscle lysates, when utrophin mRNA was normalized to the housekeeping gene Ywhaz. N = 4. *P<0.05, *P*<0.001 compared to wild-type. ^###^ *P*<0.001 compared to *mdx^4cv^*.

Utrophin expression is found on the sarcolemma of all developing wild-type muscle fibers and subsequently becomes restricted to the neuromuscular junctions [21], [54]. The prevention of skeletal muscle necrosis in the dko mice implied that the developmental loss of utrophin expression from the extrasynaptic sarcolemma did not occur. Furthermore, the expression of utrophin on the extrasynaptic sarcolemma of a portion of dko fibers suggests that utrophin may be expressed in certain muscle fiber types. To test this, we compared the expression of the utrophin A isoform relative to muscle fiber types at 3 weeks of age (Fig. 4). We found that utrophin was near absent from the extrasynaptic sarcolemma of wild-type gastrocnemius muscles by 3 weeks of age (Fig. 4). We found utrophin in the cytoplasm of a portion of the wild-type fast 2b fibers (Fig. 4). Furthermore, antibodies to the utrophin A isoform labeled blood vessels in wild-type muscles at 3 weeks of age (Fig. 4), but not at 11 weeks of age (Fig. 3), which was similar to the immunohistochemical staining pattern of the utrophin A isoform in humans [55]. Utrophin expression was absent from the extrasynaptic sarcolemma in most fast 2b fibers in desmin^−/−^, *mdx^4cv^* and dko muscles (Fig. 4). However, utrophin remained at low levels on the sarcolemma of 1a, 2a and 2d/x fiber types in desmin^−/−^ and *mdx^4cv^* gastrocnemius muscles. The reduced utrophin expression in the extrasynaptic sarcolemma of *mdx^4cv^* muscles coincided with the appearance of patches of necrotic fibers (Fig. 4). In contrast, utrophin prevented skeletal muscle necrosis in the dko muscles by remaining on the extrasynaptic sarcolemma of maturing 1a, 2a and 2d/x fiber-types (Fig. 4). We next performed a titration of utrophin by western analyses of the 3-week-old dko muscles to generate a non-linear regression to quantitate the changes in utrophin expression (Fig. S2). We found a 29.6% increase in utrophin in the *mdx^4cv^* muscles compared to wild-type controls (Fig. 4B; *P*<0.05). Utrophin in the dko was increased by a further 60.9% compared to the *mdx^4cv^* muscles (*P*<0.001). Similar to 11 weeks of age (Fig. 3D), we found no change in the relative amounts of mRNA at 3 weeks of age when comparing all genotypes (Fig. 4C). Thus, utrophin expression was increased in the dko in a fiber-type specific manner to prevent necrosis.

### Utrophin protects the sarcolemma of 1a, 2a and 2d/x dko skeletal muscle fiber types

**Figure 5:**
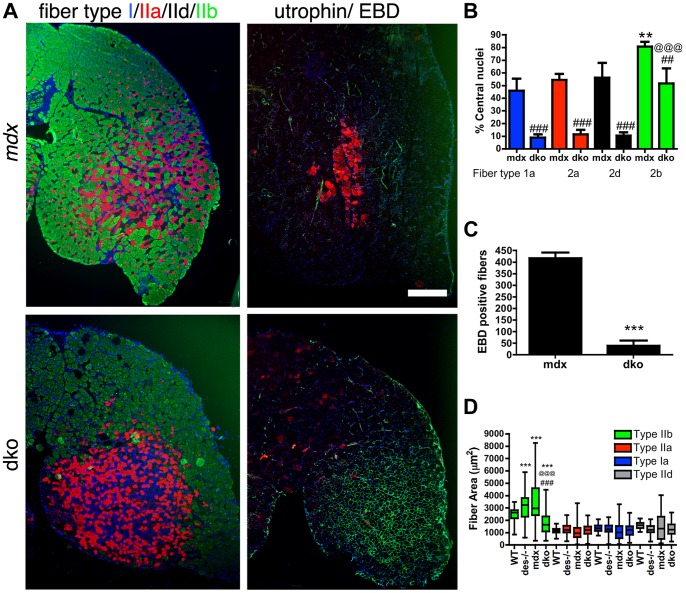
Utrophin maintains the integrity of the dko muscle membrane in a fiber-type specific manner. **A**) Shown are frozen sections of the lateral portion of the gastrocnemius muscle immunolabeled with monoclonal antibodies to fiber types 1a (blue), 2a (red), 2d/x (black) and 2b (green; left panel) or utrophin (green; right panel) and Evan's blue dye (EBD; red; right panel). Note that the uneven distribution of utrophin expression in the *mdx^4cv^* muscles correlated with patches of adjacent membrane permeable fibers that labeled with EBD. However, an increase in utrophin expression in the dko myofibers excluded EBD from the 1a, 2a and 2d/x fiber types. The dko fast 2b fibers, which lacked utrophin, were permeable to EBD. Scale bar  = 500 µm. **B**) Bars show the mean +/− S.D. percentage of centrally nucleated fibers in distinct fiber types. Note that all dko muscle fiber types had significantly less myonuclei than the *mdx^4cv^* fibers (^##^ *P*<0.01 and ^###^ *P*<0.001). The dko fast 2b fibers had more central nuclei than the 1a, 2a and 2d/x fiber types (^@@@^P<0.001). The *mdx^4cv^* fast 2b fibers had more central nuclei than the 1a, 2a and 2d/x fiber types (**P<0.01). **C**) Bars show the mean +/− S.D. total number of EBD positive fibers in the gastrocnemius muscles. ***P<0.001 compared with *mdx^4cv^* myofibers. **D**) Bars show the mean +/− S.D. area of type 1a, 2a, 2d/x and 2b muscle fiber types. ***P<0.001 compared with wild-type myofibers. ^###^ *P*<0.001 compared with *mdx^4cv^* myofibers. ^@@@^P<0.001 compared with desmin^−/−^ myofibers. All experiments were from n = 4 mice.

To examine whether utrophin prevented necrosis by maintaining the integrity of the muscle membrane, we systemically delivered 200 µl of 1% (*w*/*v*) Evan's blue dye (EBD) into the *mdx^4cv^* and dko mice and looked for permeable skeletal muscle fibers (Fig. 5A). We found large patches of skeletal muscle fibers in the *mdx^4cv^* mice that were permeable to EBD (Fig. 5A), as previously described [40]. Utrophin was selectively expressed in the dko 1a, 2a and 2d/x fiber types and prevented the infiltration of EBD into these fibers (Fig. 5A). This correlated with an ∼80% reduction in centrally nucleated 1a, 2a and 2d/x fiber types in the dko compared to the corresponding *mdx^4cv^* muscles (*P*<0.001; Fig. 5B). Only the fast 2b fibers in the dko were permeable to EBD, which correlated with an ∼5 fold increase in centrally nucleated 2b fibers when compared with the other fiber-types in the dko (*P*<0.001; Fig. 5B). The total number of permeable fibers in the dko gastrocnemius muscles was ∼91% less than the *mdx^4cv^* muscles (Fig. 5C; *P*<0.001). Thus, utrophin prevented necrosis in the dko 1a, 2a and 2d/x fiber types by maintaining the integrity of the membrane.

We found a distinct separation of the fast 2b fibers from the 1a, 2a and 2d/x fiber types in the dko gastrocnemius muscles suggestive of a fiber-type switch in the dko muscles (Fig. 5A). We examined the fiber-type proportions in the smaller soleus muscle that contains all fiber-types in wild-type C57Bl/6mice. Analysis of fiber-type proportions in the soleus muscles at 11 weeks of age revealed a significant shift from the 2a fibers in the wild-type toward the slow 1a fibers in the desmin^−/−^, *mdx^4cv^* and dko muscles (*P*<0.001; Fig. S3). However, we found no significant change in fiber-type proportions when comparing between the desmin^−/−^, *mdx^4cv^* and dko muscles (Fig. S3). Thus, the skeletal muscle fiber-types were redistributed in the dko muscles, but we found no evidence of a fiber-type switch.

The increase in utrophin on the dko sarcolemma (Fig. 3C) may have resulted from reduced surface area of the 1a, 2a and 2d/x fibers compared with the corresponding *mdx^4cv^* muscles. However, the fiber area of 1a, 2a and 2d/x fiber types within the gastrocnemius muscles was unchanged when comparing wild-type, desmin^−/−^, *mdx^4cv^* and dko muscles (Fig. 5D). The fast 2b fibers in the desmin^−/−^ and *mdx^4cv^* gastrocnemius were hypertrophic when compared to wild-type muscles (Fig. 5D). In contrast, the fast 2b fibers in the dko muscles were selectively atrophic. The desmin^−/−^ muscles contained some smaller caliber fibers that increased the overall variability in muscle fiber area. The muscle fiber areas were highly variable in the *mdx^4cv^* muscles. Thus, the increase in utrophin expression on the dko sarcolemma did not result from changes in the average area of 1a, 2a and 2d/x fiber types.

### Utrophin-independent mechanisms influence dystrophic pathology in the dko muscles

**Figure 6:**
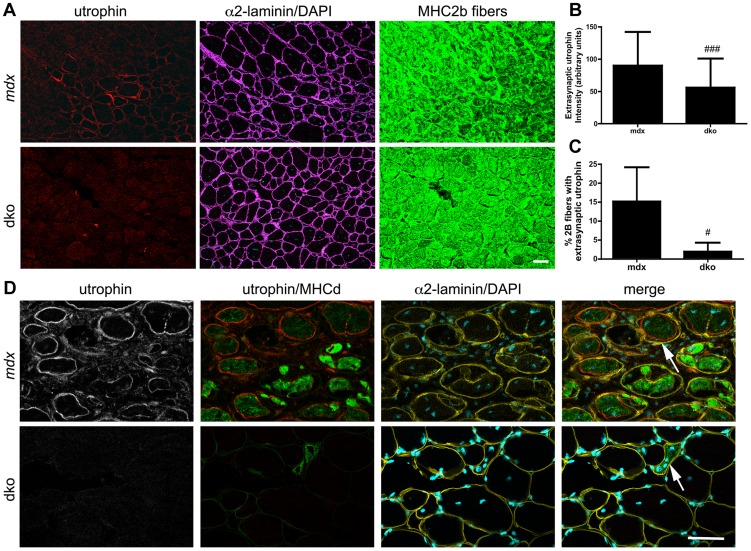
Utrophin expression was reduced on the extrasynaptic sarcolemma of dko fast 2b fibers. **A**) Utrophin expression in transverse sections of the near pure population of fast 2b fibers in the most superficial region of the gastrocnemius muscles. Shown are single sections from *mdx^4cv^* or dko gastrocnemius fast 2b fibers labeled with utrophin in red, α2-laminin in magenta, DAPI in cyan and fast 2b fibers in green. **B**) Quantitation of maximal extrasynaptic utrophin fluorescence intensity in fast 2b fibers and **C**) the proportion of fast 2b fibers expressing extrasynaptic utrophin in the *mdx^4cv^* and dko. All bars represent the mean +/− S.D. from n = 4 mice. ^#^ *P*<0.05 and ^###^ *P*<0.001 compared with *mdx^4cv^* fibers. **D**) Regenerating fibers expressed utrophin in the *mdx^4cv^* fibers, but not in the dko. Shown are single sections from *mdx^4cv^* or dko superficial gastrocnemius muscles labeled with utrophin in red, developmental myosin heavy chain in green, α2-laminin in yellow and DAPI in cyan. Note that utrophin is on the expanding sarcolemma in *mdx^4cv^* muscles, but not in the dko (arrows). Scale bars  = 50 µm.

We also found a 36% reduction in the proportion of centrally nucleated fast 2b fibers in the dko when compared to the *mdx^4cv^* fast 2b fibers (*P*<0.01; Fig. 5B), which was consistent with the low level of central nuclei in utrophin negative fibers in the dko (46%) compared to all *mdx^4cv^* control fibers (76%) (Fig. 3E). To directly test whether utrophin-independent mechanisms were influencing the dystrophic pathology we performed a more detailed examination of the most superficial region of the gastrocnemius muscles that contained a near pure population of fast 2b fibers (Fig. 6). We found a significant reduction in the extrasynaptic utrophin expression on the fast 2b fibers in the dko compared with *mdx^4cv^* muscles (Fig. 6A,B; *P*<0.001). Moreover, there was a significant reduction in the number of fast 2b fibers expressing extrasynaptic utrophin in the dko when compared to the *mdx^4cv^* fast 2b fibers (Fig. 6A,C; *P*<0.05). Utrophin was expressed on the extrasynaptic sarcolemma in groups of regenerating *mdx^4cv^* 2b fibers as the myofibers expanded toward the basal lamina shell (Fig. 6D). Utrophin expression was maintained on the *mdx^4cv^* sarcolemma as the muscles matured and developmental myosin heavy chain dissipated (Fig. 6D). In contrast, examination of four dko gastrocnemius muscles revealed that the regenerating 2b fibers were directly enveloped by the basal lamina rather than utrophin (Fig. 6D). Together, these results demonstrate that utrophin expression was reduced in the extrasynaptic sarcolemma of dko fast 2b fibers. Thus, utrophin-independent mechanisms were also mitigating the dystrophic pathology of dko muscles.

### Regenerative potential of skeletal muscles

**Figure 7:**
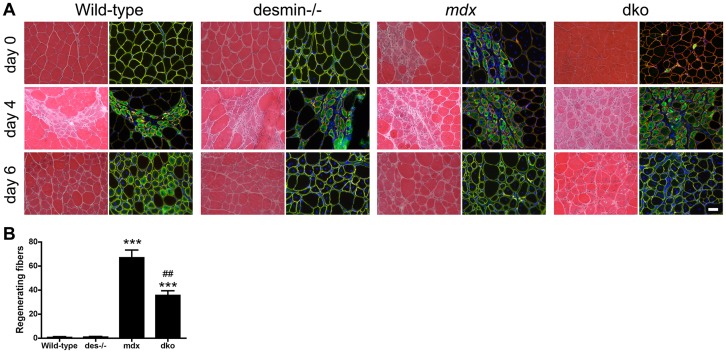
Regenerative capacity of muscle. **A**) Transverse sections of uninjured (Day 0) or injured gastrocnemius muscles 4 days and 6 days post notexin administration. Sections stained with hematoxylin and eosin are shown in the left columns and developmental myosin heavy chain (green), α2-laminin (red) and DAPI (blue) are shown in the right columns. Scale bar  = 50 µm. **B**) Bars represent the mean +/− S.D. of the total number of regenerating fibers in the uninjured gastrocnemius muscles (Day 0). ***P<0.001 compared with wild-type muscles and ^##^ *P*<0.01 compared with *mdx^4cv^* muscles. N = 4 mice for all experiments.

The regenerative capacity of skeletal muscles depleted of desmin is profoundly impaired in cell culture [56], [57]. However, muscle generation in desmin^−/−^ skeletal muscles *in vivo* is apparently normal [58]. Desmin^−/−^ muscles injured with cardiotoxin can lead to persistent expression of developmental myosin heavy chain [59]. We found that regenerating fibers in uninjured gastrocnemius muscles were rare (up to 2 fibers) in the wild-type and desmin^−/−^ mice (Fig. 7A,B). The *mdx^4cv^* muscles contained patches of regenerating fibers (Fig. 7). However, the dko muscles contained 47% fewer regenerating fibers than the *mdx^4cv^* muscles (*P*<0.01; Fig. 7A,B). To examine whether the regenerative capacity of muscles was impaired in the dko we delivered notexin to injure the gastrocnemius muscles and examined the muscles 4 and 6 days post injury. We found that regenerating fibers were expressing developmental myosin in wild-type, desmin^−/−^, *mdx^4cv^* and dko treated muscles at 4 days post injury (Fig. 7). At 6 days post injury we found that half (2 out of 4) of the injured wild-type muscles expressed developmental myosin (Fig. 7). Neither the desmin^−/−^, *mdx^4cv^* or dko muscles expressed developmental myosin 6 days post notexin injury (Fig. 7). We found no other overt changes in the regenerative capacity of the muscles when comparing the different strains of mice (Fig. 7). Thus, the improved dystrophic pathology in the dko muscles did not result from overt changes to the regenerative capacity of the skeletal muscles.

### Utrophin concentrated β-dystroglycan in the sarcolemma, but not the nNOS, α-dystrobrevin and α1-syntrophin (NODS) complex in the dko mice

**Figure 8:**
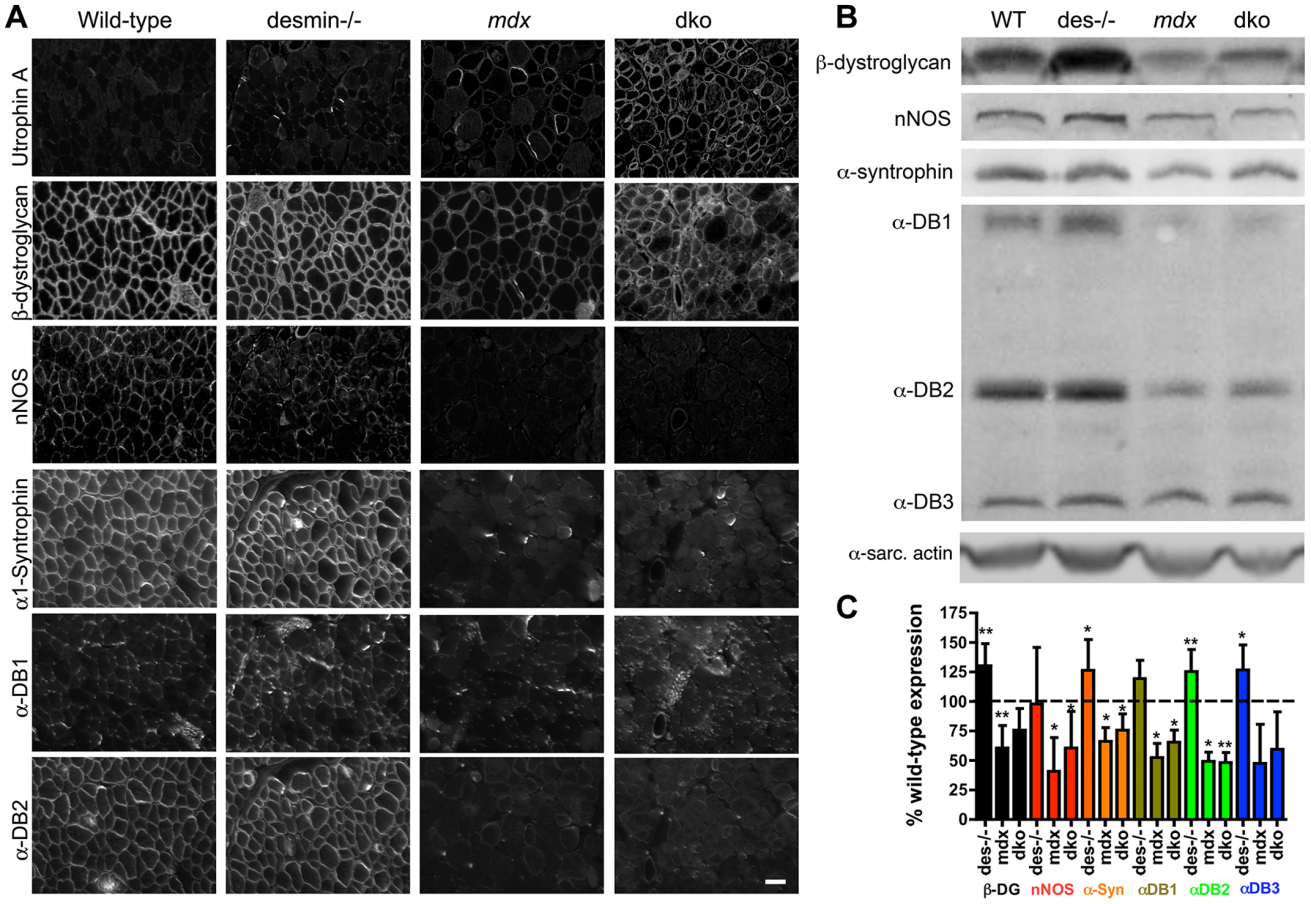
Localization and expression of β-dystroglycan and the NODS complex in skeletal muscles. **A**) Adjacent sections of gastrocnemius muscles showing the localization of β-dystroglycan and the NODS complex at the sarcolemma of wild-type, desmin^-/-^, *mdx^4cv^* and dko skeletal muscles. Scale bar  = 50 µm. **B**) Western analysis of quadriceps muscles reveals an increase in expression of DGC proteins in the desmin^-/-^ and a reduction in the *mdx^4cv^* and dko muscles. α-DB is α-dystrobrevin and α-sarc. actin is the α-sarcomeric actin loading control. **C**) Bars show mean +/− S.D. densitometric quantitation of protein expression graphed as a percentage of wild-type. *P<0.05, **P<0.01 compared to wild-type. N = 4–8 for all experiments.

We next examined whether the significant increase in utrophin expression in the dko muscles restored the expression of β-dystroglycan and the NODS complex to the sarcolemma. Adjacent sections of gastrocnemius muscles revealed that β-dystroglycan and members of the NODS complex were concentrated within the sarcolemma of wild-type and desmin^-/-^ skeletal muscles (Fig. 8A). The expression of β-dystroglycan and the NODS complex were increased in the desmin^-/-^ mice (Fig. 8B,C), as previously described [60]. The expression of β-dystroglycan and the NODS complex at the sarcolemma of *mdx^4cv^* skeletal muscles were significantly diminished (Fig. 8), as previously described [40], [61] (Fig. 8). The increase in utrophin expression in the dko sarcolemma was accompanied by the increased concentration of β-dystroglycan (Fig. 8A). Immunoblots of whole muscle lysates revealed no significant difference in β-dystroglycan expression when comparing the wild-type or the *mdx^4cv^* controls with the dko (Fig. 8B,C). However, the expression of the NODS complex on the sarcolemma of dko muscles was not restored (Fig. 8).

Desmin can interact with α-dystrobrevin in the NODS complex indirectly through synemin, syncoilin and dysbindin [46]. Therefore, we examined whether desmin expression influenced the restoration of the NODS complex (Fig. S4). We found that utrophin was expressed on the sarcolemma of 4-week-old *mdx^4cv^* soleus muscles with minimal expression of the NODS complex (Fig. S4). Thus, the lack of the NODS complex on the sarcolemma of dko skeletal muscle fibers did not result from the absence of desmin.

### Structural/functional changes in dko skeletal muscles

**Figure 9:**
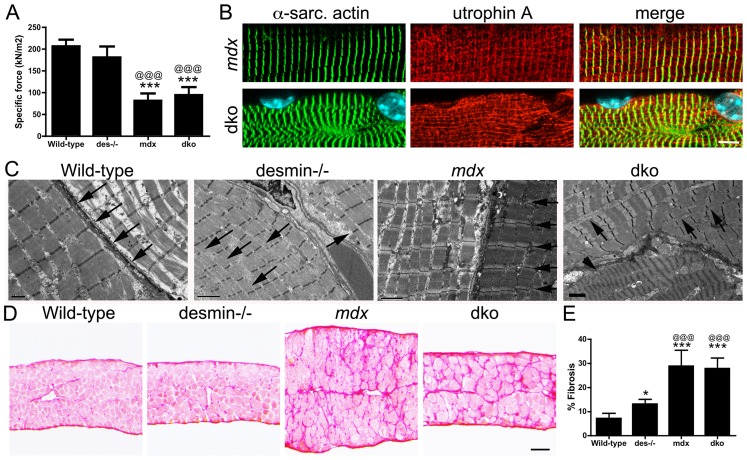
Impaired diaphragm function in the dko correlates with loss of sarcomere alignment and deposition of collagen. **A**) Mean +/− S.D. specific force of diaphragm strips in vitro from wild-type (n = 6), desmin^−/−^ (n = 5), *mdx^4cv^* (n = 5) and dko (n = 5) mice. **B**) Utrophin A colocalizes with α-sarcomeric actin in longitudinal sections. Note the misalignment of α-sarcomeric actin and utrophin A in the dko myofiber. Scale bar  = 6 µm. **C**) Electron microscopy of longitudinal sections of diaphragm muscle demonstrating the alignment of sarcomeres in wild-type and *mdx* mice (arrows). Note the alignment of sarcomeres is perturbed in desmin^−/−^ muscles (arrows) and severely impaired in the dko muscles (arrows point to misalignment of sarcomeres while the arrow head points toward a hyper-contracted myofiber). Scale bars  = 2 µm. **D**) Sirius red staining of collagen in transverse frozen sections of the wild-type (n = 8), desmin^−/−^ (n = 9), *mdx^4cv^* (n = 5) and dko (n = 5) diaphragms. Scale bar  = 100 µm. **E**) Mean +/− S.D. of Sirius red staining as a proportion of the muscle area. *P<0.05, ***P<0.001 compared to wild-type, ^@@@^P<0.001 compared to desmin^−/−^.

We next examined whether diaphragm function in the dko was influenced by structural defects within and around the muscles. We measured the specific contractile force of diaphragm strips *in vitro*. We found that the specific force production of the desmin^−/−^ diaphragm was similar to wild-type at 11 weeks of age (Fig. 9A). In contrast, the specific force production of both *mdx^4cv^* and dko diaphragms were significantly diminished (Fig. 9A; *P*<0.001). Detailed histological analyses of the *mdx^4cv^* and dko diaphragms revealed that utrophin colocalized with α-sarcomeric actin in a costameric lattice (Fig. 9B). However, the alignment of α-sarcomeric actin in the dko was severely perturbed similar to the rectilinear pattern of utrophin (Fig. 9B). Electron microscopy analyses revealed that the sarcomeres aligned in wild-type muscles, but this alignment was impaired in desmin^−/−^ muscles (Fig. 9C), as previously described [44], [45]. The alignment of sarcomeres in *mdx^4cv^* myofibers was similar to wild-type (Fig. 9C). However, the alignment of sarcomeres in the dko was severely impaired within and between individual muscle fibers (Fig. 9C). Gross histological analyses of the diaphragm revealed a 1.83-fold increase in the deposition of collagen in desmin^−/−^ compared to wild-type (*P*<0.05; Fig. 9D,E). The *mdx^4cv^* diaphragms were significantly larger and contained proportionally more collagen than wild-type (4.05-fold increase; *P*<0.001) and desmin^−/−^ controls (2.21-fold increase; *P*<0.001; Fig. 9D,E). The dko diaphragm was similar in size to the wild-type and desmin^−/−^ controls (Fig. 9D), but contained proportionately similar amounts of collagen as the *mdx^4cv^* diaphragm (28% in the dko compared to 29% in *mdx^4cv^*; Fig. 9D,E). Together, these results demonstrate that the impaired respiratory function in the dko mice resulted, at least in part, from the impaired alignment of sarcomeres and deposition of collagen between the myofibers in the diaphragm.

## Discussion

Increasing utrophin expression is a promising target for treatment of DMD [33]. While the downstream signaling pathways that influence utrophin expression are well described [33], [38], [39], the upstream mechanisms are less clear. Here, we found that perturbing the highly ordered structure of striated muscle by genetically deleting desmin from *mdx^4cv^* mice increased utrophin expression to levels that prevented skeletal muscle necrosis. We report a ∼2.5-fold increase in utrophin expression in the dko sarcolemma of 1a, 2a and 2d/x fiber types, which prevented necrosis by maintaining the integrity of the sarcolemma. Understanding the structural mechanisms that influence utrophin expression in the dko skeletal muscles may contribute to development of a therapy for DMD.

### Potential mechanisms that influence utrophin expression in the dko muscles

We found that the onset of necrosis in the *mdx^4cv^* gastrocnemius muscles was coincident with the loss of utrophin expression from the maturing fibers (Fig. 4), as previously described [22], [36]. MyoD initiates skeletal muscle differentiation and maturation by activating many skeletal muscle genes and suppressing others [62]. MyoD activates the transcription of miR-206, which targets the utrophin mRNA for degradation leading to the loss of utrophin expression from the sarcolemma and its replacement by dystrophin [63]. Analysis of C2C12 cells suggests that several other miRNAs may also repress the expression of utrophin [64]. The loss of utrophin expression from the sarcolemma of maturing fibers was delayed in desmin^−/−^ muscles and prevented in the dko muscles. It will be interesting to test whether desmin can influence the expression, trafficking, or function of miRNA's that knock-down utrophin expression.

An alternate possibility is that an early pulse in utrophin transcription [65] increased utrophin expression to levels that could overcome the knockdown effects of the miRNA's. Muscle contraction can change the shape of nuclei [66], which can change gene expression [67]–[69]. Desmin interacts with myonuclei via plectin and lamin A/C [70]–[72]. The myonuclei in the desmin^−/−^ muscles remain oval shaped in response to muscle contraction [66]. This could potentially lead to the persistence of a developmental gene expression program that underlies the increased utrophin expression in the dko.

Utrophin is normally expressed at low levels on the sarcolemma of the slower oxidative fibers in wild-type mice [73]. Inducing the oxidative myogenic program can alleviate the dystrophic pathology in *mdx* mice by stimulating utrophin expression. For instance, activation of PGC1α [74]–[76], calcineurin A/NFAT [77]–[80], GA binding protein [74], Ca2+/calmodulin [81], AMP activated protein kinase [82], and the transcriptional activator PPARβ/δ [83] can each induce the slow oxidative program in *mdx* muscle and increase utrophin expression. Metabolic changes to the muscle can also influence utrophin expression [84]. While we found no significant change in fiber-types when comparing *mdx*, desmin^−/−^ and dko soleus muscles (Fig. S3), we did find utrophin expression on the extrasynaptic sarcolemma of 1a, 2a and 2d/x fiber-types, but not in the fast 2b fibers. Thus, our results are consistent with the activation of the slower oxidative myogenic pathways that can induce utrophin expression.

The absence of desmin in stressed muscle is associated with a shift in the expression of muscle proteins to those found in slow-twitch fibers [85], [86]. These changes may be mediated in part by changes in the activity of calcineurin linked to alter myoplasmic Ca^2+^ levels, which could result from a loss of local protein kinase A (PKA) signaling linked to the loss of desmin. The copolymerization of desmin with synemin in the intermediate filament reticulum contributes to synemin's localization around Z-disks [87], [88]. As synemin is an A kinase anchor protein (AKAP) [89] the absence of desmin in the dko is likely to alter local PKA activity associated with the sarcomere. Calcium homeostasis is likely to be affected locally as PKA can regulate many channels and transporters essential for normal excitation-contraction coupling [90]–[93]. However, our finding that the mRNA levels for utrophin do not change in extracts of dko gastrocnemius muscle, compared to age-matched wild-type, desmin^−/−^ and *mdx^4cv^* muscles, argue against this mechanism.

While there are various signaling pathways that can activate utrophin transcription in *mdx* mice, we found no changes in utrophin mRNA in the dko total gastrocnemius muscle lysates when compared to the *mdx^4cv^*, desmin^−/−^ or wild-type muscles. The persistence of utrophin on the dko sarcolemma of maturing skeletal muscle fibers is consistent with increased utrophin stability and post-transcriptional mechanisms. Proteins experimentally over-expressed within the *mdx* extrasynaptic sarcolemma such as sarcospan [94], [95], cytotoxic T cell GalNac transferase [96] and biglycan [35] can stabilize utrophin to prevent skeletal muscle necrosis. RhoA, a small GTPase also increases utrophin expression without apparently influencing transcription [97]. Stabilizing RNA, a known function for the type III intermediate filament protein vimentin [98], is another potential mechanism that can increase utrophin expression without changing transcription [99], [100]. Desmin may also influence protein degradation pathways by trafficking lysosomes through the muscle via its interaction with myospryn [101], [102].

### The lack of NODS expression may impair the therapeutic efficacy of utrophin in the dko

Increasing utrophin expression by increasing utrophin transcription or stabilization can restore the expression of the DGC to the sarcolemma [53], [96], [103]–[105], except for nNOS [106]. We found that utrophin was able to concentrate β-dystroglycan to the sarcolemma in the dko 1a, 2a and 2d/x fiber types. However, the expression of the NODS sub-complex was not restored in the dko muscles. nNOS influences blood flow to the skeletal muscles and can lead to hypoxic stress injury post-exercise [12]. However, the long-term effects of the lack of nNOS are difficult to predict considering Becker muscular dystrophy patients expressing truncated dystrophins can have a mild phenotype without restoring nNOS to the sarcolemma [12], [107]. The low level of α-dystrobrevin on the sarcolemma may have contributed to the low level of central nuclei in the dko mice, as α-dystrobrevin^−/−^ mice have a mild dystrophy and residual expression of α2-dystrobrevin mitigates the dystrophic pathology in *mdx* muscles [13]. While α1-syntrophin is an important adapter protein that is required for the localization of nNOS and aquaporin to the sarcolemma of striated muscle [108], [109], its role in the pathogenesis of DMD is unclear. The low level of the NODS complex in the dko muscles did not result from the lack of desmin (Fig. S4). Thus, the low level of central nuclei (∼9%) in the dko muscle fibers with extrasynaptic utrophin likely resulted from the lack of desmin in combination with the reduced expression of the NODS complex from the extrasynaptic sarcolemma.

### Utrophin-independent mechanisms influence dystrophic pathology

We found that the dystrophic pathology in the fast 2b fibers was also improved in the dko despite a significant reduction in extrasynaptic utrophin expression when compared with *mdx^4cv^* fast 2b fibers. Most striking was the fact that utrophin expression was reduced in the extrasynaptic sarcolemma of regenerating fast 2b fibers in the dko. However, we found no overt change in the regenerative capacity of the muscle stem cells in the dko gastrocnemius muscles injured with notexin. In contrast, Agbulut and colleagues found that desmin^−/−^ muscles injured with cardiotoxin displayed persistent expression of developmental myosin, small caliber fibers and the infiltration of adipocytes [59]. Here, we found no evidence of increased adipocytes in the desmin^−/−^ or dko muscles. Therefore, the discrepancy between our studies may have resulted from the different myotoxins. In any case, we found a significant reduction in the number of necrotic fibers in the dko supporting a mechanism that prevents dystrophy rather than influencing regeneration. Desmin is also likely to play a structural role in linking the contractile apparatus to the sarcolemma [47], [52], [101] and in regulating the passive mechanical properties of skeletal muscle [66], [110]. We found that utrophin could form costameric striations with α-sarcomeric actin in dko mice, but the rectilinear pattern was severely impaired. The exacerbated loss of sarcomere alignment in dko diaphragms suggests the absence of desmin and potentially the NODS complex could weaken the sarcomeric connections to the membrane. However, it is important to note that the specific force production of *mdx^4cv^* and dko diaphragms was comparable. The *mdx^4cv^* mice have a compensatory hypertrophy that can potentially maintain peak force production [111]. However, the dko diaphragms lack this cellular hypertrophy suggesting that the impaired diaphragm function could contribute to the respiratory distress and shortened lifespan. Considering the dko mice die prematurely from apparent cardiorespiratory failure, it is possible that reduced mobility in the cage could mitigate contraction-induced injury to the muscles. We are currently investigating whether desmin influences contraction-induced injury to the sarcolemma in *mdx^4cv^* muscles.

### Conclusion

In conclusion, we report a significant increase in utrophin expression in dko skeletal muscles that prevented necrosis in a fiber-type specific manner. The fact that utrophin expression was elevated ∼2.5-fold on the dko sarcolemma when compared with *mdx^4cv^* muscles is of considerable interest for developing treatments for DMD [26]. Clearly, deleting desmin is not a therapeutic option for DMD as the dko mice die from apparent cardiorespiratory distress, but understanding the upstream mechanisms that influence utrophin expression may lead to novel treatment strategies for DMD. Furthermore, an utrophin-mediated therapy developed from the dko mice would treat all muscle fiber-types in the human as humans lack the fast 2b fiber types. Considering desmin functions to maintain the highly ordered structure of striated muscles [44], [45], it is likely that utrophin expression in the dko is initiated by changes to muscle structure/signaling relationships. We also found that utrophin-independent mechanisms were improving the dystrophic pathology in dko fast 2b fibers, which will be of interest for understanding the pathophysiology of DMD. Thus, the dko mice may provide new insights into the regulation of utrophin expression that are relevant for the treatment of DMD.

## Materials and Methods

### Mice and ethics statement

We utilized C57Bl/6 wild-type mice, desmin^−/−^ mice, *mdx^4cv^* mice and *mdx*:desmin dko mice. All experiments were in accordance with the Institute of Animal Care and Use Committee of the University of Washington. The desmin^−/−^ mice were a kind gift from Professor Yassemi Capetanaki. We generated the dko mice by first backcrossing the desmin^−/−^ mice from the FVB strain to the wild-type C57Bl/6 strain for five generations (N5). The resulting desmin^−/−^ mice on the C57Bl/6 strain were then inbred for at least four generations to obtain desmin^−/−^ controls (>F4) or they were crossed with the *mdx^4cv^* strain on the C57Bl/6 background and inbred for at least four generations to obtain the dko mice (>F4). Therefore, the mice generated for this study were B6.FVB-*Desmin* and B6.FVB-*Desmin*-*mdx^4cv^* incipient congenic with ∼96.9% homozygosity with the C57Bl/6 background. We genotyped the mice using standard PCR for desmin and performed sequence analysis of the *mdx^4cv^* genomic DNA to avoid potential false positives as previously described [112]. The desmin^−/−^ and dko mice were sacrificed if they lost body mass or exhibited signs of cardiorespiratory distress. Kaplan-Meyer survival analysis was performed with 16 desmin^−/−^ male mice and 13 dko male mice.

### Diaphragm function

The diaphragm physiology was performed as previously described [113]. Briefly, the diaphragm from wild-type (n = 6), desmin^−/−^ (n = 5), *mdx^4cv^* (n = 5) and dko (n = 5) was placed in oxygenated KREBS (2 mM Ca^2+^, 24 mM NaHCO_3_, 137 mM NaCl, 5 mM KCl, 1 mM MgSO_4_, 1 mM NaH_2_PO_4_, D-Glucose). Strips of the diaphragm were dissected and the optimum length and peak tetanic contractile force was measured over 350 ms. Because the diaphragm strips vary in size, a direct comparison of peak contractile force is not plausible. After contraction, the diaphragm strip is weighed and specific force was calculated as peak tetanic force production × length × density (1.04) × pennation (1 for the diaphragm)/muscle mass.

### Costamere analysis

Costamere analysis was performed as previously described [52]. Briefly, the mice were anaesthetized with 2,2,2-tribromoethanol (Sigma) and perfused with 2% paraformaldehyde (Electron microscopy sciences). The muscles were incubated in 2% paraformaldehyde for 2 hours at 4°C, then washed 3 times with 1× PBS, and incubated in 10% sucrose for 1 hour at 4°C, and then 20% sucrose overnight at 4°C. The muscles were then placed in cryovials and flash frozen in liquid N_2_. The frozen samples were placed on a frozen chuck with OCT and 40 µm thick sections were cut using a cryostat. The sections were immunostained with 1∶800 utrophin A polyclonal antibody (kind gift from Stanley Froehner) and 1∶500 α-sarcomeric actin monoclonal antibody (SIGMA). The thick sections were imaged using a Leica SP5 confocal microscope.

### Electron microscopy

The electron microscopy was performed on longitudinal sections of diaphragm muscle as previously described [114].

### Histology

Muscles were frozen directly in OCT cooled in 2-methylbutane in liquid N_2_. Ten micrometer transverse sections of skeletal muscles were stained with hematoxylin and eosin, alizarin red and Sirius red using manufacturer protocols (Electron Microscopy Sciences; Hatfeild, PA). The Sirius red staining of collagen was measured using the manufacturers protocols in Image J analyses software. Transverse frozen sections were also immunostained as previously described [40]. Briefly, the sections were incubated in blocking buffer (1% BSA, 0.05% Triton X-100 in 1× phosphate buffered saline (PBS)) for 30 minutes and immunostained with antibodies to desmin (1∶50; DAKO Corp), N-terminal dystrophin antibody (1∶800), utrophin (1∶800), α-dystrobrevin 1 (1: 500), α-dystrobrevin 2 (1∶1000), α1-syntrophin (1∶500; the latter four antibodies were kind gifts from Stanley C. Froehner), β-dystroglycan (1∶100; Transduction Laboratories), MHCd (1∶40; Novocastra), α2-laminin (1∶800; Sigma) or nNOS (Zymed; 1∶100) for 1 hour. The sections were washed 3 times in 1× PBS for 10 minutes each and incubated in Alexa-488, Alexa-555, Alexa-594 or Alexa-647 secondary antibodies for 30 minutes (1∶800; Invitrogen). To label necrotic fibers we immunostained the muscles with mouse IgG_1_ antibodies conjugated to Alexa 488 (1∶800; Invitrogen). For labeling of acetylcholine receptors we incubated the sections in α-bungarotoxin conjugated to TRITC for 1 hour (1∶800; Invitrogen). The sections were washed 3 times for 10 minutes each and coverslipped with ProLong Gold mounting medium containing DAPI (Invitrogen). Muscle fiber typing was performed using conjugated monoclonal antibodies as previously described [115]. Sections were imaged with either a Leica SP5 confocal (Fig. 1, 3, 6), Nikon eclipse E1000 (Fig. 2, 7, 8) or an Olympus SZX16 dissection fluorescent microscope (Fig. 4, 5).

### Quantitation of utrophin staining of muscle sections

Quantitation of maximal sarcolemmal utrophin fluorescence intensity was performed as previously described for dystrophin [116]. Briefly, gastrocnemius muscle sections and images were processed identically for quantitation. We utilized the FIJI analyses software to quantitate maximal fluorescence intensity. The utrophin fluorescence intensity on the wild-type sarcolemmal was used as a negative control and the utrophin fluorescence intensity at the wild-type synapse was used as the peak of detection. We drew a line across the images to ensure unbiased quantitation and measured the peak fluorescent intensity that coincided with extrasynaptic sarcolemma staining. The sarcolemmal utrophin fluorescence intensity from *mdx^4cv^*, dko and microutrophin^ΔR4–R21^ treated *mdx*∶utrophin double knockout muscles all fell within these limits. The mean +/− S.D. fluorescence intensity from n = 4 mice from 92 wild-type, 99 desmin^−/−^, 100 *mdx^4cv^*, 112 dko, and 77 microutrophin^ΔR4–R21^ treated *mdx*:utrophin double knockout myofibers were compared.

### Evans blue dye

The *mdx^4cv^* and dko mice (n = 4) were administered 200 µl of 0.22 µm filter sterilized 1% (*w*/*v*) EBD solution in HBSS intravenously by retro-orbital injection. Mice were sacrificed 3 hours after EBD administration. The gastrocnemius muscles were frozen in OCT in 2-methylbutane in liquid N_2_. Ten micrometer sections were cut and stained for utrophin (1∶800; kind gift from Stanley Froehner). Utrophin was labeled with Alexa-488 goat anti-rabbit secondary antibody (Invitrogen). The sections were viewed and imaged using the Olympus SZX16 dissection fluorescent microscope.

### Muscle fiber regeneration

The gastrocnemius muscles of wild-type, desmin^−/−^, *mdx^4cv^* and dko (n = 8) were administered 30 µl of 1 µg/ml notexin in PBS at 11 weeks of age. The mice were sacrificed 4 days (n = 4) and 6 days (n = 4) post-injury. The gastrocnemius muscles were frozen in OCT. Ten micrometer sections were immunostained with α2-laminin (1∶800; Sigma) and developmental myosin heavy chain (1∶40; Novocastra) and directly compared to adjacent sections stained with hematoxylin and eosin. Considering monoclonal antibodies can label necrotic fibers, we defined regenerating fibers as those fibers that expressed developmental myosin heavy chain and contained centrally located nuclei.

### Immunoblotting

Western blots were performed on whole muscle lysates as previously described [40]. Briefly, the gastrocnemius muscles of 3 and 11-week-old wild-type, desmin^−/−^, *mdx^4cv^* and dko (n = 6) were ground in liquid N_2_ and homogenized in extract buffer (50 mM Tris-HCl, 150 mM NaCl, 0.2% SDS, 24 mM Na Deoxycholate, 1% NP40, 47.6 mM Na Fluoride, 200 mM Na Orthovanadate, Roche). Protein concentration of whole muscle was determined by Coomassie Plus Bradford Assay (Pierce). Equal amounts of protein (10 µg) were resolved on a 4–12% SDS polyacrylamide gel. The blots were incubated in utrophin (1∶1000; kind gift from Stanley C. Froehner) overnight at 4°C. The α-sarcomeric actin primary antibody (1∶500; Sigma) was used as a loading control as its expression was unchanged when comparing the different strains of mice, as previously described for wild-type versus *mdx^4cv^* [40], [117]. We also loaded 20 µg of total protein to compare the expression of desmin, β-dystroglycan (1∶100; BD Transduction laboratories), α1-syntrophin (1∶500; kind gift from Stanley C. Froehner), pan α-dystrobrevin (1∶1000; BD Transduction laboratories) primary antibodies. The primary antibodies were detected with IgG HRP secondary antibodies (1∶6000; Jackson ImmunoResearch Labs). The blots were developed with ECL plus (Pierce) and scanned with the Storm 860 imaging system (Amersham Biosciences). The band intensity was measured using Image J software (NIH). The relative amount of utrophin in each blot was determined using a non-linear regression generated by a titration of utrophin from the dko from 1.25 µg up to 20 µg of total loaded protein and examined using the PRISM statistics software (Figures S1, S2; n = 4 for wild-type and desmin^−/−^ and n = 8 for *mdx^4cv^* and dko samples).

### Real time PCR

To isolate the RNA, approximately 20 µg of gastrocnemius muscle previously ground by mortar and pestle in liquid N_2_ was used to extract total RNA following manufacturers instructions (TRI Reagent, Molecular Research Center). We used gastrocnemius muscles from 11 week old (Fig. 3D) or 3 week old mice (Fig. 4C). The pelleted RNA was suspended in 50 µl nuclease free elution solution (Ambion, Austin, TX). Five µg of total RNA was treated with Turbo DNA-free (Ambion, Austin, TX) in order to remove trace amounts of contaminating DNA. The DNAase Treated RNA (0.5 µg) was diluted to 8 µl with nuclease free water followed by use of the SuperScript™ III First-Strand Synthesis kit (Invitrogen, Carlsbad, CA) to generate cDNA. Subsequently 2 µl of the cDNA was used for qPCR with utrophin primer-probe sets. The mouse utrophin primers sequences were: Forward 5′- ACCAGCTGGACCGATGGA-3′, Reverse 5′- CTCGTCCCAGTCGAAGAGATCT-3′, Probe 5′-6FAM- CGTTCAACGCCGTGCTCCACC-3′-BHQa1-Q. As a reference gene the oligonucleotide set was used to target the mouse Ywhaz gene sequence (Tyrosine 3-monooxygenase; [118]): Forward 5′- GCTGGTGATGACAAGAAAGGAAT-3′, Reverse 5′- GGTGTGTCGGCTGCATCTC-3′, Probe 5′-6FAM- TGGACCAGTCACAGCAAGCATACCAAGA-3′-BHQa1-Q.

### Statistics

The data were compared using a one-way ANOVA with a Tukey post-test that compares all data sets with a Student's t-test. The relative amounts of utrophin in western analyses were determined using a non-linear regression generated from a titration of utrophin in the dko gastrocnemius muscles (from 1.25 µg–20 µg of total added protein). All data analyses were performed using the PRISM software.

## Supporting Information

Figure S1 **A**) Western analyses demonstrating a titration of utrophin and α-sarcomeric actin in n = 4, 11-week-old dko gastrocnemius muscles. **B**) Relative amounts of utrophin detected compared to the total amount of protein loaded onto the blots.
(TIF)

Figure S2 **A**) Western analyses demonstrating a titration of utrophin and α-sarcomeric actin in n = 4, 3-week-old dko gastrocnemius muscles. **B**) Relative amounts of utrophin detected compared to the total amount of protein loaded onto the blots.
(TIF)

Figure S3 Graph shows the mean +/− S.D. percentage of muscle fiber types in the soleus muscles. There were significantly more slow 1a fibers in the desmin^−/−^ (n = 4), *^mdx4cv^* (n = 3) and dko soleus (n = 4) muscles when compared with the wild-type muscles (n = 3) ***P<0.001. There were also significantly fewer 2a fibers in the in the desmin^−/−^, *mdx^4cv^* and dko soleus muscles when compared with wild-type muscles ^#^ *P*<0.05; ^##^ *P*<0.01; ^###^ *P*<0.001.
(TIF)

Figure S4 Desmin expression did not influence restoration of the NODS complex on the sarcolemma. **A**) Note that utrophin was expressed in the sarcolemma of *mdx^4cv^* soleus muscles with desmin at 4 weeks of age, but **B**) did not restore α1-syntrophin, α-dystrobrevin 1 or α-dystrobrevin 2 localization. Scale bar  = 50 µm.
(TIF)
