# Supplementary figures and images for: Muscle Structure Influences Utrophin Expression in mdx Mice
Source: PLoS Genet. 2014 Jun 12;10(6):e1004431. doi: 10.1371/journal.pgen.1004431 (PMC4055409; doi:10.1371/journal.pgen.1004431)

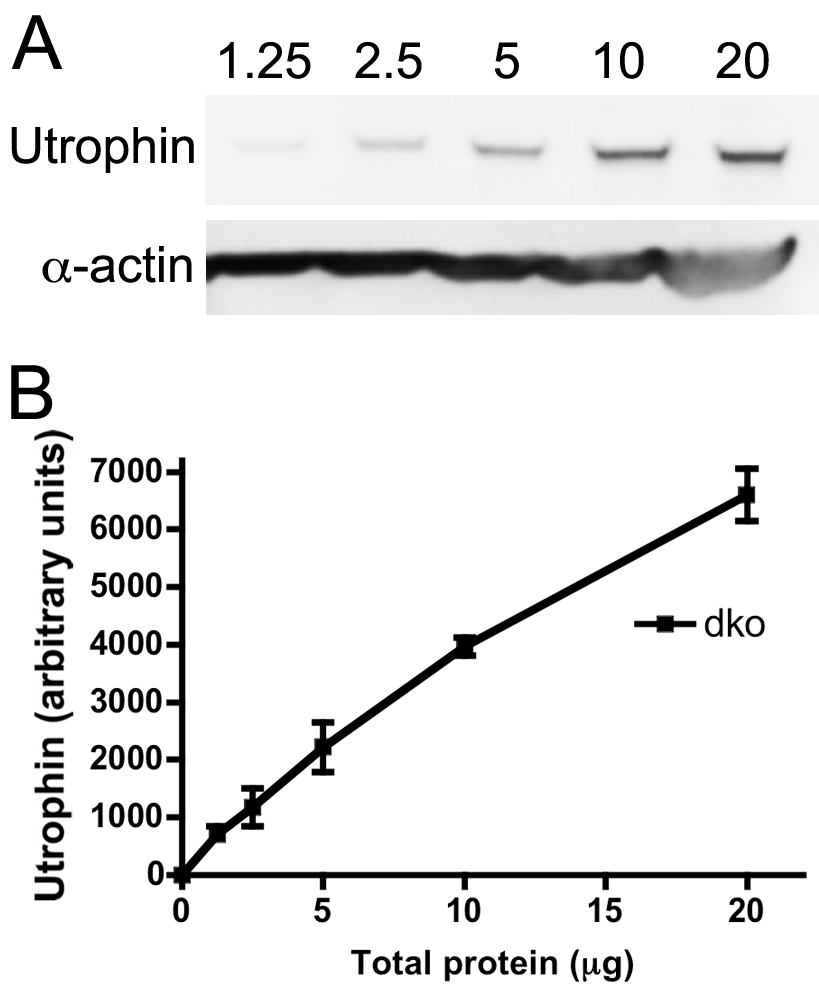

Supplement: Figure S1 — A) Western analyses demonstrating a titration of utrophin and α-sarcomeric actin in n = 4, 11-week-old dko gastrocnemius muscles. B) Relative amounts of utrophin detected compared to the total amount of protein loaded onto the blots. (TIF) [file pgen.1004431.s001.tif]

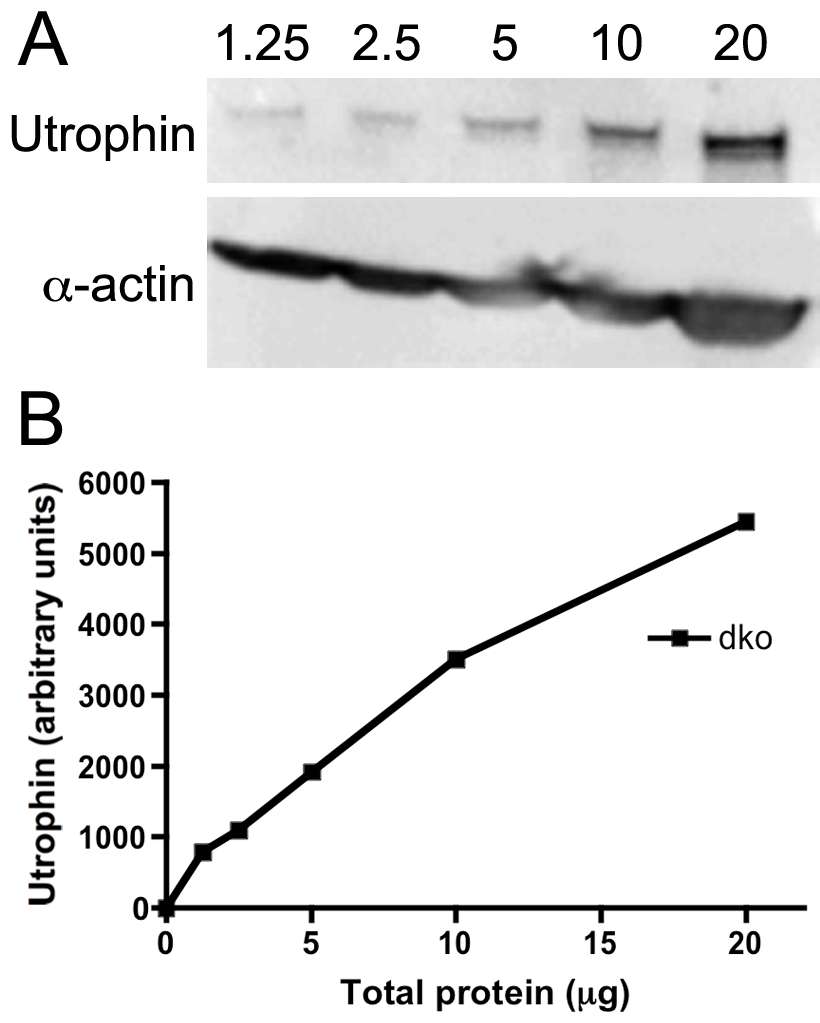

Supplement: Figure S2 — A) Western analyses demonstrating a titration of utrophin and α-sarcomeric actin in n = 4, 3-week-old dko gastrocnemius muscles. B) Relative amounts of utrophin detected compared to the total amount of protein loaded onto the blots. (TIF) [file pgen.1004431.s002.tif]

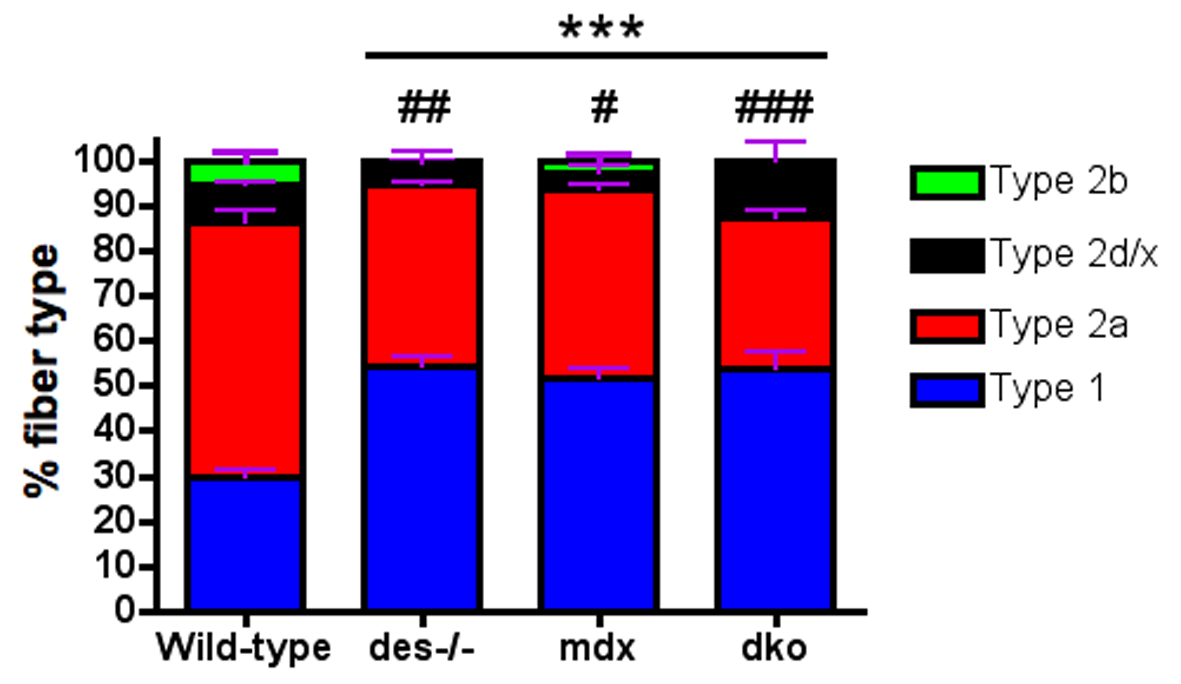

Supplement: Figure S3 — Graph shows the mean +/− S.D. percentage of muscle fiber types in the soleus muscles. There were significantly more slow 1a fibers in the desmin−/− (n = 4), mdx4cv (n = 3) and dko soleus (n = 4) muscles when compared with the wild-type muscles (n = 3) ***P<0.001. There were also significantly fewer 2a fibers in the in the desmin−/−, mdx4cv and dko soleus muscles when compared with wild-type muscles # P<0.05; ## P<0.01; ### P<0.001. (TIF) [file pgen.1004431.s003.tif]

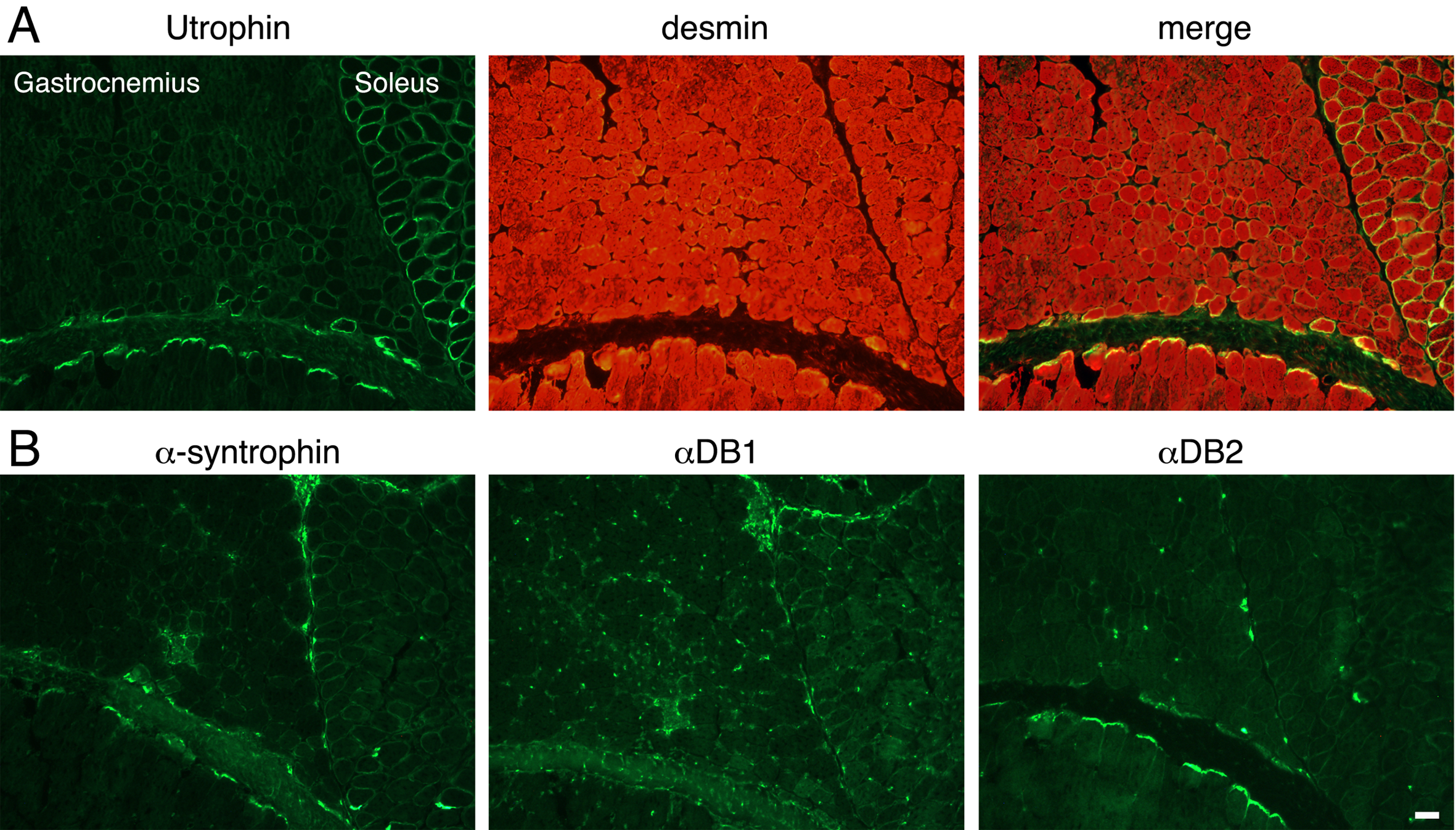

Supplement: Figure S4 — Desmin expression did not influence restoration of the NODS complex on the sarcolemma. A) Note that utrophin was expressed in the sarcolemma of mdx4cv soleus muscles with desmin at 4 weeks of age, but B) did not restore α1-syntrophin, α-dystrobrevin 1 or α-dystrobrevin 2 localization. Scale bar = 50 µm. (TIF) [file pgen.1004431.s004.tif]
